# Real-World Survival Outcomes Associated With First-Line Immunotherapy, Targeted Therapy, and Combination Therapy for Metastatic Clear Cell Renal Cell Carcinoma

**DOI:** 10.1001/jamanetworkopen.2021.11329

**Published:** 2021-05-25

**Authors:** Nicholas H. Chakiryan, Da David Jiang, Kyle A. Gillis, Elizabeth Green, Ali Hajiran, Lee Hugar, Logan Zemp, Jingsong Zhang, Rohit K. Jain, Jad Chahoud, Philippe E. Spiess, Wade Sexton, Scott M. Gilbert, Brandon J. Manley

**Affiliations:** 1 Department of Genitourinary Oncology, H. Lee Moffitt Cancer Center and Research Institute, Tampa, Florida; 2 Department of Urology, Beth Israel Deaconess Medical Center, Boston, Massachusetts; 3 Department of Urology, University of Iowa Hospitals & Clinics, Iowa City

## Abstract

**Importance:**

Clinical trials have shown an overall survival (OS) benefit associated with first-line immunotherapy (IT) and combination targeted therapy (TT) and IT regimens compared with TT among patients with metastatic clear cell renal cell carcinoma (RCC). Generalizability of these findings in a real-world cohort outside of a clinical trial setting is unclear.

**Objective:**

To assess the association of first-line TT, IT, and combination TT and IT regimens with OS in a real-world cohort of patients with metastatic clear cell RCC.

**Design, Setting, and Participants:**

This retrospective propensity-matched cohort study identified 5872 patients with metastatic clear cell RCC in the National Cancer Database from January 1, 2015, to December 31, 2017, who received first-line TT, IT, or combination TT and IT and were not treated on a clinical trial protocol. Patients were stratified by first-line systemic treatment. Statistical analysis was conducted from October 1 to December 1, 2020.

**Main Outcomes and Measures:**

The primary outcome was OS from the date of diagnosis to death or censoring at last follow-up. After 1:1:1 nearest-neighbor caliper matching of propensity scores, survival analyses were conducted using Cox proportional hazards regression and Kaplan-Meier estimates.

**Results:**

The final study population included 5872 patients (TT group: n = 4755 [81%]; 3332 men [70%]; median age, 64 years [interquartile range, 57-71 years]; IT group: n = 638 [11%]; 475 men [74%]; median age, 61 years [interquartile range, 54-69 years]; and combination TT and IT group: n = 479 [8%]; 321 men [67%]; median age, 62 years [interquartile range, 55-69 years]), and the matched cohort included 1437 patients (479 per treatment group). Patients in the IT and combination TT and IT groups were younger than those in the TT group, had fewer comorbid conditions (Charlson-Deyo score of 0, 480 of 638 [75%] in the TT group, 356 of 479 [74%] in the IT group, and 3273 of 4755 [69%] in the combination TT and IT group), and were more often treated at academic centers (315 of 638 [49%], 216 of 479 [45%], and 1935 of 4755 [41%], respectively). Both first-line IT and combination TT and IT were associated with improved OS compared with first-line TT for patients with metastatic clear cell RCC (IT group: hazard ratio [HR], 0.60 [95% CI, 0.48-0.75]; *P* < .001; combination TT and IT group: HR, 0.74 [95% CI, 0.60-0.91]; *P* = .005). No survival difference was seen between the IT and combination TT and IT groups (combination TT and IT: HR, 1.24 [95% CI, 0.98-1.56]; *P* = .08).

**Conclusions and Relevance:**

This study suggests that both first-line IT and combination TT and IT were associated with improved OS compared with first-line TT for patients with metastatic clear cell RCC. These findings are similar to those identified in recently reported clinical trials, lending confidence to the broader applicability of these findings outside of a clinical trial setting.

## Key Points

**Question:**

What is the comparative effectiveness of first-line targeted therapy, immunotherapy, and combination therapy in a real-world cohort of patients with metastatic clear cell renal cell carcinoma?

**Findings:**

In this propensity-matched cohort study of 5872 patients treated in real-world clinical practice, first-line immunotherapy and combination therapy were associated with improved overall survival compared with first-line targeted therapy.

**Meaning:**

This study suggests that clinical trial findings demonstrating an overall survival benefit associated with first-line immunotherapy-based regimens compared with targeted therapy are generalizable to a broader population of patients.

## Teaser

This cohort study assesses the association of first-line targeted therapy, immunotherapy, and combination targeted therapy and immunotherapy regimens with overall survival in a real-world cohort of patients with metastatic clear cell renal cell carcinoma (ccRCC).

## Introduction

Renal cell carcinoma (RCC) is the sixth most common cancer among men and the eighth most common cancer among women in the US, accounting for 4.2% of all incident cancer cases and 2.4% of all cancer deaths each year.^1^ Approximately 30% of patients with RCC present with either regional or distant metastases, and 20% of individuals who receive an initial diagnosis of localized disease will eventually develop regional or distant metastases.^1,2,3^ Clear cell RCC is the most common histologic subtype, representing approximately 80% of all RCCs.^4^

First-line management of metastatic clear cell RCC has recently shifted toward immunotherapy (IT), largely owing to the emergence of immune checkpoint blockade (ICB) therapies and clinical trial results demonstrating improved overall survival (OS) and progression-free survival compared with tyrosine kinase–inhibiting targeted therapy (TT).^5,6,7,8^ Current guideline recommendations for preferred first-line IT-based therapy for metastatic clear cell RCC include both dual IT (eg, ipilimumab plus nivolumab) and combination TT and IT (eg, axitinib plus pembrolizumab) regimens.^9^ Although the safety and efficacy of IT-based regimens have been demonstrated in clinical trials, to our knowledge, their effectiveness among more generalizable populations has not yet been assessed. Evaluating new treatments in comparative effectiveness studies is a critical part of validating clinical trial findings because patients enrolled in clinical trials tend to be younger and healthier than those encountered in real-world practice.^10,11,12^ Given the limited availability of effectiveness data for novel and increasingly used IT regimens for metastatic clear cell RCC, we sought to examine real-world survival outcomes for TT, IT, and combination TT and IT regimens using a generalizable cohort of patients with metastatic clear cell RCC.

## Methods

### Study Population and Data Source

Cases of clear cell RCC were identified and abstracted from the National Cancer Database (NCDB) between January 1, 2015, and December 31, 2017. The NCDB includes more than 70% of cancer cases diagnosed in the United States, which are reported by member facilities of the Commission on Cancer. These facilities are not limited to academic centers, with more than 50% of participating facilities representing community cancer programs or comprehensive community cancer programs.^13^ Trained data abstractors collect and submit data to the NCDB using standardized coding definitions as specified in the most recent Commission on Cancer Facility Oncology Registry Data Standards guideline.^14^ This study was conducted using deidentified data and was determined to be exempt by the H. Lee Moffitt Cancer Center and Research Institute institutional review board because the analysis exclusively involved deidentified patient data and is considered a secondary analysis of existing data. This study was reported in a manner consistent with the Strengthening the Reporting of Observational Studies in Epidemiology (STROBE) reporting guideline.^15^

The year 2015 was chosen as the earliest date for the analysis because it was the first year of US Food and Drug Administration (FDA) approval for an ICB agent (nivolumab, as second-line therapy) for the treatment of metastatic clear cell RCC.^8,16^ Criteria for case inclusion were clinical stage IV metastatic clear cell RCC at diagnosis, age between 18 and 100 years, availability of complete staging and demographic data, and receipt of either IT, TT, or combination TT and IT as first-line treatments. Patients in the TT and IT groups received those therapies alone. Cases were excluded if NCDB codes indicated that the patient was treated on an experimental or blinded clinical trial protocol.

### Variables and Definitions

Consistent with previously peer-reviewed and published NCDB studies regarding TT use in RCC, TT was defined as the receipt of single or multiagent systemic chemotherapy.^17,18,19,20^ Immunotherapy was defined as the receipt of a first-line systemic IT regimen. Immunotherapy is defined in the NCDB using the SEER*Rx Interactive Drug Database.^21^ Thus, patients in the IT cohort may have received non-ICB regimens, such as interferon α or high-dose interleukin 2. Combination TT and IT was defined as concurrent receipt of both systemic TT and systemic IT as first-line therapy. Age was defined as the age at initial diagnosis. Comorbidities were measured according to the Charlson-Deyo method and scored as discrete count categories (0, 1, 2, or ≥3) per NCDB reporting standards.^22,23^ Overall survival was measured from the date of initial diagnosis to the date of death or censorship at last follow-up.

### Statistical Analysis

Statistical analysis was conducted from October 1 to December 1, 2020. Patients were stratified into groups based on receipt of TT, IT, or combination TT and IT as first-line treatment. Baseline patient and tumor characteristics were abstracted from the data set. Analysis of variance testing was used to compare continuous variables, the χ^2^ test of independence was used to compare normally distributed categorical variables, and the Kruskal-Wallis test was used to compare nonnormally distributed categorical variables.

Propensity score matching using a 1:1:1 nearest-neighbor match without replacement was performed with a caliper width of 0.2 SDs of the propensity score distribution, as previously described.^24,25^ Baseline variables used to formulate propensity scores included age, sex, race/ethnicity, Charlson-Deyo score, facility type, insurance status, year of diagnosis, cT stage, cN stage, and cytoreductive nephrectomy status. Univariable analyses were repeated in the postmatching cohorts to assess for resolution of statistically significant differences between groups.

The a priori design for the survival analysis was set to include any variables from the postmatching univariable analysis with *P* ≤ .10 as covariates in a multivariable Cox proportional hazards regression for OS and, if none of the variables met these criteria, to then perform a univariable Cox proportional hazards regression for OS stratified by treatment group. Two Cox proportional hazards regressions were used, 1 with TT as the reference in the therapy group and 1 with IT as the reference, to allow for 3 direct pairwise comparisons between treatment groups. A sensitivity analysis was then performed using a multivariable Cox proportional hazards regression for OS, including all patient and tumor demographic variables as covariates. Kaplan-Meier estimates were used to generate survival functions, and a log-rank test for equality of survivor functions was used.

The primary outcome involved 3 comparisons, and to reduce the risk of making a type I error, the definition of statistical significance for the Cox proportional hazards regression was adjusted using Bonferroni correction and defined as a 2-tailed α risk of .0167 or less.^26^ All other analyses maintained the standard definition of statistical significance as a 2-tailed α risk of .05 or less. All statistical analyses and data visualization were performed using the R program, version 4.0.2 (R Project for Statistical Computing). Propensity matching was performed using the MatchIt package for R, and survival analyses were performed using the survival and survminer packages for R.

## Results

### Study Population

**Figure 1.**
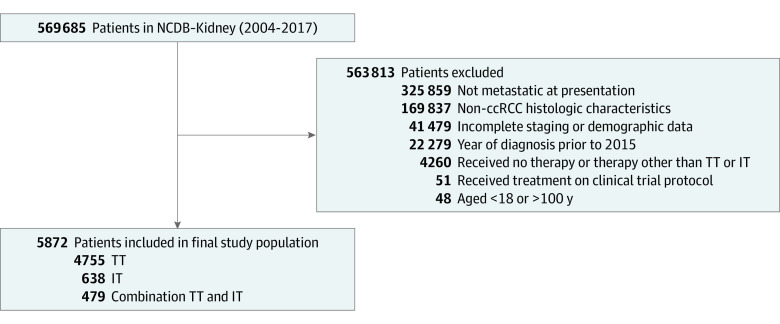
Flow Diagram Detailing Selection of the Study Population ccRCC indicates clear cell renal cell carcinoma; IT, immunotherapy; NCDB, National Cancer Data Base; and TT, targeted therapy.

We identified 569 685 patients in the NCDB with a malignant neoplasm of the kidney or renal pelvis, and after application of the inclusion and exclusion criteria, the final study population included 5872 patients, with 4755 patients (81%) having received first-line TT (median age, 64 years [interquartile range, 57-71 years]; 3332 male patients [70%]; 4123 White patients [87%]), 638 patients (11%) having received first-line IT (median age, 61 years [interquartile range, 54-69 years]; 475 male patients [74%]; 563 White patients [88%]), and 479 patients (8%) having received first-line combination TT and IT (median age, 62 years [interquartile range, 55-69 years]; 321 male patients [67%]; 421 White patients [88%]) (Table 1). A flow diagram for cohort selection is outlined in Figure 1. Complete baseline demographic and clinical characteristics are shown in Table 1.

**Table 1.** Prematching Patient and Tumor Characteristics, Baseline and Pathologic, by Therapy Type

Characteristic	No. (%)			*P* value^a^
	TT (n = 4755)	IT (n = 638)	Combination TT and IT (n = 479)	
Age, median (IQR), y	64 (57-71)	61 (54-69)	62 (55-69)	<.001
Sex				
Male	3332 (70)	475 (74)	321 (67)	.02
Female	1423 (30)	163 (26)	158 (33)	
Race/ethnicity				
White	4123 (87)	563 (88)	421 (88)	.30
Black	403 (9)	41 (6)	40 (8)	
Other^b^	229 (5)	34 (5)	18 (4)	
Charlson-Deyo comorbidities, No.				
0	3273 (69)	480 (75)	356 (74)	.001
1	896 (19)	94 (15)	73 (15)	
2	320 (7)	38 (6)	37 (8)	
≥3	266 (6)	26 (4)	13 (3)	
Facility type				
Academic	1935 (41)	315 (49)	216 (45)	<.001
Nonacademic	2820 (59)	323 (51)	263 (55)	
Insurance				
Not insured	156 (3)	16 (3)	10 (2)	<.001
Private insurance	1956 (41)	330 (52)	240 (50)	
Medicaid	411 (9)	45 (7)	36 (8)	
Medicare	2111 (44)	229 (36)	184 (38)	
Other government	57 (1)	7 (1)	5 (1)	
Unknown	64 (1)	11 (2)	4 (1)	
Year of diagnosis				
2015	1537 (32)	136 (21)	126 (26)	<.001
2016	1641 (35)	186 (29)	162 (34)	
2017	1577 (33)	316 (50)	191 (40)	
cT stage				
cT1	1306 (27)	145 (23)	132 (28)	.30
cT2	1603 (34)	234 (37)	160 (33)	
cT3	1386 (29)	199 (31)	137 (29)	
cT4	460 (10)	60 (9)	50 (10)	
cN stage				
cN0	3034 (64)	434 (68)	319 (67)	.07
cN+	1721 (36)	204 (32)	160 (33)	
Cytoreductive nephrectomy	1798 (38)	335 (53)	201 (42)	<.001

Abbreviations: IQR, interquartile range; IT, immunotherapy; TT, targeted therapy.

^a^ Statistical tests performed included Kruskal-Wallis test, χ^2^ test of independence, and analysis of variance.

^b^ Other race/ethnicity included American Indian, Aleutian, or Eskimo; Chinese; Japanese; Filipino; Hawaiian; Korean; Vietnamese; Laotian; Hmong; Kampuchean (including Khmer and Cambodian); Thai; Asian Indian or Pakistani, not otherwise specified; Asian Indian; Pakistani; Micronesian; Chamorran; Guamanian, not otherwise specified; Polynesian, not otherwise specified; Tahitian; Samoan; Tongan; Melanesian, not otherwise specified; Fiji Islander; New Guinean; other Asian, including Asian, not otherwise specified, and Oriental, not otherwise specified; Pacific Islander, not otherwise specified; and other race/ethnicity.

The incidence of treatment with TT decreased during the period studied (2015: 85% [1537 of 1799]; 2016, 83% [1641 of 1989]; 2017: 76% [1577 of 2084]), and treatment with either IT or combination TT and IT increased (2015: 15% [262 of 1799]; 2016, 18% [348 of 1989]; 2017: 24% [507 of 2084]) (eFigure in the Supplement). The median time from diagnosis to initiation of therapy was 49 days (interquartile range, 27-79 days).

In 1:1:1 nearest-neighbor caliper matching without replacement, the smallest group, combination TT and IT, was considered the “treatment” group. The matching algorithm successfully matched each patient from the combination TT and IT group with a counterpart from each of the other 2 groups. The postmatching population included 1437 patients, with 479 in each treatment group. Postmatching univariable analyses confirmed resolution of statistically significant differences between groups for all variables included in the propensity matching (Table 2).

**Table 2.** Postmatching Patient and Tumor Characteristics, Baseline and Pathologic, by Therapy Type

Characteristic	No. (%)			*P* value^a^
	TT (n = 479)	IT (n = 479)	Combination TT and IT (n = 479)	
Age, median, (IQR), y	61 (53-68)	61 (55-69)	62 (55-69)	.40
Sex				
Male	335 (70)	333 (70)	321 (67)	.60
Female	144 (30)	146 (30)	158 (33)	
Race/ethnicity				
White	420 (88)	428 (89)	421 (88)	>.99
Black	42 (9)	35 (7)	40 (8)	
Other^b^	17 (4)	16 (3)	18 (4)	
Charlson-Deyo comorbidities, No.				
0	356 (74)	359 (75)	356 (74)	>.99
1	77 (16)	75 (16)	73 (15)	
2	34 (7)	33 (7)	37 (8)	
≥3	12 (3)	12 (3)	13 (3)	
Facility type				
Academic	219 (46)	227 (47)	216 (45)	.80
Nonacademic	260 (54)	252 (53)	263 (55)	
Insurance				
Not insured	20 (4)	13 (3)	10 (2)	.70
Private insurance	216 (45)	244 (51)	240 (50)	
Medicaid	44 (9)	33 (7)	36 (8)	
Medicare	182 (38)	175 (37)	184 (38)	
Other government	6 (1)	4 (1)	5 (1)	
Unknown	11 (2)	10 (2)	4 (1)	
Year of diagnosis				
2015	138 (29)	125 (26)	126 (26)	.40
2016	164 (34)	148 (31)	162 (34)	
2017	177 (37)	206 (43)	191 (40)	
cT stage				
cT1	155 (32)	135 (28)	132 (28)	.70
cT2	149 (31)	167 (35)	160 (33)	
cT3	124 (26)	127 (27)	137 (29)	
cT4	51 (11)	50 (10)	50 (10)	
cN stage				
cN0	312 (65)	314 (66)	319 (67)	.90
cN+	167 (35)	165 (34)	160 (33)	
Cytoreductive nephrectomy	187 (39)	218 (46)	201 (42)	.13

Abbreviations: IQR, interquartile range; IT, immunotherapy; TT, targeted therapy.

^a^ Statistical tests performed included Kruskal-Wallis test, χ^2^ test of independence, and analysis of variance.

^b^ Other race/ethnicity included American Indian, Aleutian, or Eskimo; Chinese; Japanese; Filipino; Hawaiian; Korean; Vietnamese; Laotian; Hmong; Kampuchean (including Khmer and Cambodian); Thai; Asian Indian or Pakistani, not otherwise specified; Asian Indian; Pakistani; Micronesian; Chamorran; Guamanian, not otherwise specified; Polynesian, not otherwise specified; Tahitian; Samoan; Tongan; Melanesian, not otherwise specified; Fiji Islander; New Guinean; other Asian, including Asian, not otherwise specified, and Oriental, not otherwise specified; Pacific Islander, not otherwise specified; and other race/ethnicity.

### Survival Analysis

Per the a priori study design, because none of the postmatching patient or tumor demographic variables demonstrated differences between groups with *P* ≤ .10, a univariable Cox proportional hazards regression for OS was performed as the primary outcome. This analysis demonstrated that both IT and combination TT and IT were associated with significantly better OS than TT for patients with metastatic clear cell RCC (IT group: hazard ratio [HR], 0.60 [95% CI, 0.48-0.75]; *P* < .001; combination TT and IT group: HR, 0.74 [95% CI, 0.60-0.91]; *P* = .005) (Table 3). With the IT group as the reference, no significant difference in OS was seen for patients who received combination TT and IT (HR, 1.24 [95% CI, 0.98-1.56]; *P* = .08). The χ^2^ testing of the Schoenfeld residuals associated with the Cox proportional hazards regression model confirmed that the proportional hazards assumption was not violated (eTable 3 in the Supplement).

**Table 3.** Univariable Cox Proportional Hazards Regressions for Overall Survival, Matched Cohort

Reference group	Comparator group	HR (95% CI)	*P* value^a^
TT	IT	0.60 (0.48-0.75)	<.001
	Combination TT and IT	0.74 (0.60-0.91)	.005
IT	Combination TT and IT	1.24 (0.98-1.56)	.08

Abbreviations: HR, hazard ratio; IT, immunotherapy; TT, targeted therapy.

^a^ Significance defined as an α risk of .0167 or less, adjusted for 3 comparisons using the Bonferroni correction.

As a sensitivity analysis, a multivariable Cox proportional hazards regression for OS was performed including all patient and tumor demographic variables as covariates (eTable 1 in the Supplement). In this analysis, both IT and combination TT and IT were associated with significantly better OS than TT (IT group: HR, 0.70 [95% CI, 0.57-0.91]; *P* = .006; combination TT and IT group: HR, 0.76 [95% CI, 0.61-0.94]; *P* = .01). With the IT group as the reference, no significant difference in OS was seen for patients who received combination TT and IT (HR, 1.06 [95% CI, 0.85-1.36]; *P* = .60).

**Figure 2.**
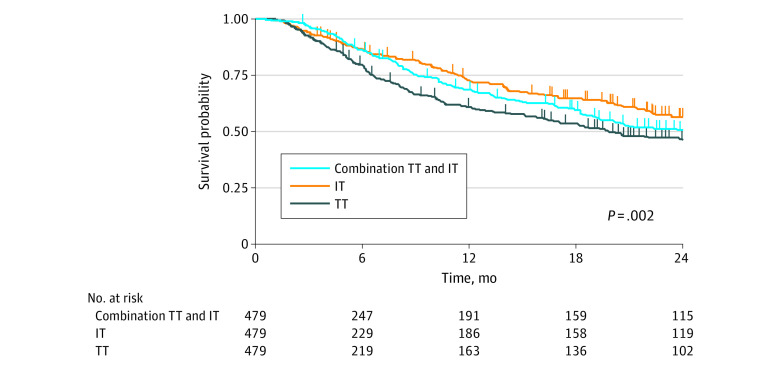
Kaplan-Meier Estimates for Overall Survival, Stratified by Treatment Group Log-rank *P* value reported within the figure. IT indicates immunotherapy; and TT, targeted therapy.

Kaplan-Meier estimates were generated to visually depict the survival distributions (Figure 2). The 12-month OS was 59% in the TT group, 73% in the IT group, and 68% in the combination TT and IT group. The median follow-up for patients who were alive at last follow-up was 9.6 months (interquartile range, 5.3-17.2 months).

## Discussion

The results of our study provide external validation for the OS benefit associated with IT and combination TT and IT regimens compared with TT alone as first-line therapy for metastatic clear cell RCC among a nationally representative cohort of patients treated in real-world clinical practice. Patients encountered in real-world clinical practice tend to be older and have more comorbidities than those enrolled in clinical trials, emphasizing the importance of studying more generalizable populations.^10,12^ A strength of the NCDB is its broad generalizability because this data set captures more than 70% of newly diagnosed cancer cases in the United States.

CheckMate 214, a landmark study comparing the dual IT regimen ipilimumab plus nivolumab with sunitinib as first-line therapy for metastatic clear cell RCC, showed a significant OS benefit for patients receiving ipilimumab plus nivolumab.^5^ In our analysis, the comparison between IT and TT confirmed this result, with a similar effect size (CheckMate 214: HR, 0.63 [95% CI, 0.44-0.89]; NCDB: HR, 0.60 [95% CI, 0.48-0.75]).

KEYNOTE-426, another landmark study comparing the combination TT and IT regimen axitinib plus pembrolizumab with sunitinib as first-line therapy for metastatic clear cell RCC, showed a significant OS benefit for patients receiving axitinib plus pembrolizumab.^7^ In our analysis, the comparison between combination TT and IT and TT confirmed this result (KEYNOTE-426: HR, 0.53 [95% CI, 0.38-0.74]; NCDB: HR, 0.74 [95% CI, 0.60-0.91]).

JAVELIN Renal 101 compared the combination TT and IT regimen axitinib plus avelumab with sunitinib as first-line therapy for metastatic clear cell RCC and did not show a significant OS difference between groups in the overall cohort; however, it did show a significant improvement in progression-free survival between groups in both the overall population and programmed death ligand 1 (PD-L1)–positive group.^6^ Because the NCDB does not report progression-free survival as an outcome or PD-L1 positivity as a pathologic variable, these specific outcomes could not be evaluated in our analysis.

The 12-month OS results reported in CheckMate 214^5^ and KEYNOTE-426^7^ were significantly higher than those identified in our analysis, across all treatment groups (eTable 2 in the Supplement). JAVELIN Renal 101 did not explicitly report 12-month OS, but inspection of the Kaplan-Meier distributions suggests values greater than 80% in both study groups.^6^ These findings confirm that patients enrolled into clinical trials tend to be at a lower overall risk of mortality than those encountered in real-world clinical practice, regardless of treatment received. This difference likely reflects the fact that clinical trial participants are carefully selected for enrollment, excluding patients with high comorbidity and poor function. Our analysis confirms that the OS benefits seen in clinical trial cohorts for IT and combination TT and IT are also evident in a cohort of patients treated in real-world clinical practice, lending confidence to the broader applicability of these findings.

Highlighting the expansion of combination TT and IT options available as first-line agents for patients with metastatic clear cell RCC, the results from 2 clinical trials have been reported in the brief time since this article was initially prepared.^27,28^ Lenvatinib plus pembrolizumab^28^ and nivolumab plus cabozantinib^27^ combinations have both demonstrated significantly improved OS compared with sunitinib alone. There is a wide array of highly effective options currently available in this disease setting.

An interesting consideration is that ICB therapy was not approved by the FDA as a first-line therapy for metastatic clear cell RCC until April 2018 (ipilimumab plus nivolumab), so any use of ICB during the period studied (2015-2017) was either via clinical trial protocol or off-label practice.^16^ Patients treated on a clinical trial or experimental protocol were excluded from this analysis, leaving us to draw the conclusion that most patients receiving IT were treated off-label. The rationale behind therapy selection is absent from the NCDB, but the prematching demographic characteristics provided in Table 1 demonstrate younger age and lower Charlson-Deyo score for patients who received IT-based regimens. Perhaps early reports of robust tumor response to IT encouraged clinicians to administer off-label first-line IT regimens to patients with fewer competing risks.

### Limitations

There are several limitations to this analysis. Primarily, there is an inherent risk of selection bias involved in retrospective comparative effectiveness studies. Despite nearest-neighbor caliper matching of propensity scores resulting in 3 groups without statistically significant differences in baseline demographic characteristics, the risk of selection bias due to unmeasured confounding will always exist in studies of this nature. In addition, the analysis was not designed to correct for immortal time bias regarding treatment effect on survival. Follow-up for the survival analysis was short (median follow-up, 9.6 months). In addition, the NCDB broadly categorizes systemic therapies such that the names of the medications are not available. Thus, patients labeled as having received IT may have received non-ICB regimens, such as interferon α, high-dose interleukin 2, or potentially misclassified non–immune-modulating monoclonal antibodies (eg, bevacizumab). These non-ICB IT regimens are seldom used as first-line therapy in the postcytokine era, and given that 20% of patients in this cohort received an IT-based regimen, with increasing incidence over time, we are reassured that most of these patients likely received ICB therapy.^29,30^ Nevertheless, for this reason, the category is labeled IT and not ICB. Likewise, specific information is lacking regarding subsequent lines of therapy beyond the first-line treatment. Finally, the NCDB does not include data on kidney function, body mass index, performance status, treatment-related toxic effects, response rates, progression-free survival, recurrence-free survival, PD-L1 status, or tumor mutational burden, all of which would have contributed to this analysis if available for study.

## Conclusions

This analysis of a nationally representative real-world cohort demonstrated that both IT and combination TT and IT were associated with improved OS for patients with metastatic clear cell RCC compared with TT alone. These findings imply the broader generalizability of previously reported clinical trial outcomes.

## Notes

Supplement. **eTable 1.** Multivariable Cox Proportional Hazards Regression for Overall Survival, Summary Table, Postmatched Cohort **eTable 2.** 12- and 18-Month OS for Patients in the Current Analysis (NCDB) and as Reported in Relevant Clinical Trials **eTable 3.** χ^2^ Statistic of the Schoenfeld Residuals for the Univariable Cox Proportional Hazards Regression Summarized in Table 3 **eFigure.** Incidence of First-Line Treatment Regimens Over Time Stratifying by TT, IT, and Combination TT/IT

## References

[1] Siegel RL, Miller KD, Jemal A. Cancer statistics, 2019. CA Cancer J Clin. 2019;69(1):7-34. doi:10.3322/caac.21551 30620402

[2] Janzen NK, Kim HL, Figlin RA, Belldegrun AS. Surveillance after radical or partial nephrectomy for localized renal cell carcinoma and management of recurrent disease. Urol Clin North Am. 2003;30(4):843-852. doi:10.1016/S0094-0143(03)00056-9 14680319

[3] Abu-Ghanem Y, Powles T, Capitanio U, . The impact of histological subtype on the incidence, timing, and patterns of recurrence in patients with renal cell carcinoma after surgery—results from RECUR Consortium. Eur Urol Oncol. Published online October 24, 2020. doi:10.1016/j.euo.2020.09.005 33109495

[4] Cairns P. Renal cell carcinoma. Cancer Biomark. 2010;9(1-6):461-473. doi:10.3233/CBM-2011-0176 22112490 PMC3308682

[5] Motzer RJ, Tannir NM, McDermott DF, ; CheckMate 214 Investigators. Nivolumab plus ipilimumab versus sunitinib in advanced renal-cell carcinoma. N Engl J Med. 2018;378(14):1277-1290. doi:10.1056/NEJMoa1712126 29562145 PMC5972549

[6] Motzer RJ, Penkov K, Haanen J, . Avelumab plus axitinib versus sunitinib for advanced renal-cell carcinoma. N Engl J Med. 2019;380(12):1103-1115. doi:10.1056/NEJMoa1816047 30779531 PMC6716603

[7] Rini BI, Plimack ER, Stus V, ; KEYNOTE-426 Investigators. Pembrolizumab plus axitinib versus sunitinib for advanced renal-cell carcinoma. N Engl J Med. 2019;380(12):1116-1127. doi:10.1056/NEJMoa1816714 30779529

[8] Motzer RJ, Escudier B, McDermott DF, ; CheckMate 025 Investigators. Nivolumab versus everolimus in advanced renal-cell carcinoma. N Engl J Med. 2015;373(19):1803-1813. doi:10.1056/NEJMoa1510665 26406148 PMC5719487

[9] Motzer RJ, Jonasch E, Michaelson MD, . NCCN guidelines insights: kidney cancer, version 2.2020. J Natl Compr Canc Netw. 2019;17(11):1278-1285. doi:10.6004/jnccn.2019.0054 31693980

[10] Elting LS, Cooksley C, Bekele BN, . Generalizability of cancer clinical trial results: prognostic differences between participants and nonparticipants. Cancer. 2006;106(11):2452-2458. doi:10.1002/cncr.21907 16639738

[11] Sherman RE, Anderson SA, Dal Pan GJ, . Real-world evidence—what is it and what can it tell us? N Engl J Med. 2016;375(23):2293-2297. doi:10.1056/NEJMsb1609216 27959688

[12] Mailankody S, Prasad V. Overall survival in cancer drug trials as a new surrogate end point for overall survival in the real world. JAMA Oncol. 2017;3(7):889-890. doi:10.1001/jamaoncol.2016.5296 27892992

[13] American College of Surgeons. About cancer program categories. Accessed December 14, 2020. https://www.facs.org/quality-programs/cancer/coc/accreditation/categories#incp

[14] Commission on Cancer. Facility oncology registry data standards: 2016. Accessed December 14, 2020. https://www.facs.org/-/media/files/quality-programs/cancer/ncdb/fords-2016.ashx

[15] von Elm E, Altman DG, Egger M, Pocock SJ, Gøtzsche PC, Vandenbroucke JP; STROBE Initiative. Strengthening the Reporting of Observational Studies in Epidemiology (STROBE) statement: guidelines for reporting observational studies. BMJ. 2007;335(7624):806-808. doi:10.1136/bmj.39335.541782.AD 17947786 PMC2034723

[16] US Food and Drug Administration. KEYTRUDA (pembrolizumab) for injection, for intravenous use. Accessed December 2, 2020. https://www.accessdata.fda.gov/drugsatfda_docs/label/2019/125514s040lbl.pdf

[17] Hanna N, Sun M, Meyer CP, . Survival analyses of patients with metastatic renal cancer treated with targeted therapy with or without cytoreductive nephrectomy: a National Cancer Data Base study. J Clin Oncol. 2016;34(27):3267-3275. doi:10.1200/JCO.2016.66.7931 27325852 PMC5024547

[18] Bhindi B, Habermann EB, Mason RJ, . Comparative survival following initial cytoreductive nephrectomy versus initial targeted therapy for metastatic renal cell carcinoma. J Urol. 2018;200(3):528-534. doi:10.1016/j.juro.2018.03.077 29574109

[19] Smaldone MC, Handorf E, Kim SP, . Temporal trends and factors associated with systemic therapy after cytoreductive nephrectomy: an analysis of the National Cancer Database. J Urol. 2015;193(4):1108-1113. doi:10.1016/j.juro.2014.10.095 25444991

[20] Chakiryan NH, Acevedo AM, Garzotto MA, . Survival outcomes and practice trends for off-label use of adjuvant targeted therapy in high-risk locoregional renal cell carcinoma. Urol Oncol. 2020;38(6):604.e1-604.e7. doi:10.1016/j.urolonc.2020.02.028 32241693

[21] National Cancer Institute, Surveillance, Epidemiology, and End Results Program. SEER*Rx interactive antineoplastic drugs database. 2020. Accessed December 14, 2020. https://seer.cancer.gov/seertools/seerrx/

[22] Charlson ME, Pompei P, Ales KL, MacKenzie CR. A new method of classifying prognostic comorbidity in longitudinal studies: development and validation. J Chronic Dis. 1987;40(5):373-383. doi:10.1016/0021-9681(87)90171-8 3558716

[23] Deyo RA, Cherkin DC, Ciol MA. Adapting a clinical comorbidity index for use with *ICD-9-CM* administrative databases. J Clin Epidemiol. 1992;45(6):613-619. doi:10.1016/0895-4356(92)90133-8 1607900

[24] Austin PC. An introduction to propensity score methods for reducing the effects of confounding in observational studies. Multivariate Behav Res. 2011;46(3):399-424. doi:10.1080/00273171.2011.568786 21818162 PMC3144483

[25] Austin PC. Optimal caliper widths for propensity-score matching when estimating differences in means and differences in proportions in observational studies. Pharm Stat. 2011;10(2):150-161. doi:10.1002/pst.433 20925139 PMC3120982

[26] Bland JM, Altman DG. Multiple significance tests: the Bonferroni method. BMJ. 1995;310(6973):170. doi:10.1136/bmj.310.6973.170 7833759 PMC2548561

[27] Choueiri TK. 696O_PR—Nivolumab + cabozantinib vs sunitinib in first-line treatment for advanced renal cell carcinoma: first results from the randomized phase III CheckMate 9ER trial. Presented at: ESMO Virtual Congress 2020; September 19, 2020; Virtual.

[28] Motzer R, Alekseev B, Rha SY, ; CLEAR Trial Investigators. Lenvatinib plus pembrolizumab or everolimus for advanced renal cell carcinoma. N Engl J Med. 2021. doi:10.1056/NEJMoa2035716 33616314

[29] Pal S, Gong J, Mhatre SK, . Real-world treatment patterns and adverse events in metastatic renal cell carcinoma from a large US claims database. BMC Cancer. 2019;19(1):548. doi:10.1186/s12885-019-5716-z 31174493 PMC6555983

[30] Hawkins R, Fife K, Hurst M, . Treatment patterns and health outcomes in metastatic renal cell carcinoma patients treated with targeted systemic therapies in the UK. BMC Cancer. 2020;20(1):670. doi:10.1186/s12885-020-07154-z 32680483 PMC7368711

