# Supplementary material for: Real-World Survival Outcomes Associated With First-Line Immunotherapy, Targeted Therapy, and Combination Therapy for Metastatic Clear Cell Renal Cell Carcinoma
Source: JAMA Netw Open. 2021 May 25;4(5):e2111329. doi: 10.1001/jamanetworkopen.2021.11329 (PMC8150693; doi:10.1001/jamanetworkopen.2021.11329)
Supplement: Supplement. — eTable 1. Multivariable Cox Proportional Hazards Regression for Overall Survival, Summary Table, Postmatched Cohort eTable 2. 12- and 18-Month OS for Patients in the Current Analysis (NCDB) and as Reported in Relevant Clinical Trials eTable 3. χ2 Statistic of the Schoenfeld Residuals for the Univariable Cox Proportional Hazards Regression Summarized in Table 3 eFigure. Incidence of First-Line Treatment Regimens Over Time Stratifying by TT, IT, and Combination TT/IT [file jamanetwopen-e2111329-s001.pdf]

Supplementary Online Content

Chakiryan NH, Jiang DD, Gillis KA, et al. Real-world survival outcomes associated with first-line immunotherapy, targeted therapy, and combination therapy for metastatic clear cell renal cell carcinoma. *JAMA Netw Open.* 2021;4(5):e2111329. doi:10.1001/jamanetworkopen.2021.11329

eTable 1. Multivariable Cox Proportional Hazards Regression for Overall Survival, Summary Table, Postmatched Cohort

eTable 2. 12- and 18-Month OS for Patients in the Current Analysis (NCDB) and as Reported in Relevant Clinical Trials

eTable 3. χ^2 Statistic of the Schoenfeld Residuals for the Univariable Cox Proportional Hazards Regression Summarized in Table 3

eFigure. Incidence of First-Line Treatment Regimens Over Time Stratifying by TT, IT, and Combination TT/IT

This supplementary material has been provided by the authors to give readers additional information about their work.

© 2021 Chakiryan NH et al. *JAMA Network Open.*

eTable 1. Multivariable Cox Proportional Hazards Regression for Overall Survival, Summary Table, Postmatched Cohort. Covariates included: age, sex, race, Charlson-Deyo score, facility type, insurance status, year of diagnosis, cT stage, cN stage, and cytoreductive nephrectomy status.

**Reference Group**	**Comparator Group**	**HR**[1]	**95% CI**[1]	***P* Value***
Targeted Therapy		—	—	—
	Immunotherapy	0.7	0.57-0.91	0.006
	Combination TT/IT	0.76	0.61-0.94	0.012
Immunotherapy		—	—	—
	Combination TT/IT	1.06	0.85-1.36	0.6

[1]*HR = Hazard Ratio, CI = Confidence Interval*

**Significance defined as α-risk ≤ 0.0167, adjusted for three comparisons using the Bonferroni correction*

© 2021 Chakiryan NH et al. *JAMA Network Open.*

eTable 2. 12- and 18-Month OS for Patients in the Current Analysis (NCDB) and as Reported in Relevant Clinical Trials

	**Overall Survival**	
	**12mo.**	**18mo.**
**NCDB**		
TT	0.59	0.46
IT	0.73	0.64
Combination TT/IT	0.68	0.59
**CheckMate-214**		
Sunitinib	0.72	0.6
Ipi/Nivo	0.8	0.75
**KEYNOTE-426**		
Sunitinib	0.78	NA
Axi/Pembro	0.9	NA

© 2021 Chakiryan NH et al. *JAMA Network Open.*

eTable 3. χ^2 Statistic of the Schoenfeld Residuals for the Univariable Cox Proportional Hazards Regression Summarized in Table 3. Figures are identical for “Therapy Group” and “GLOBAL”, as this was a univariable regression with “Therapy Group” being the sole variable.

	**Chisq**	**Df**	***P* Value**
**Therapy Group**	3.03	2	0.22
**GLOBAL**	3.03	2	0.22

© 2021 Chakiryan NH et al. *JAMA Network Open.*

eFigure. Incidence of First-Line Treatment Regimens Over Time Stratifying by TT, IT, and Combination TT/IT

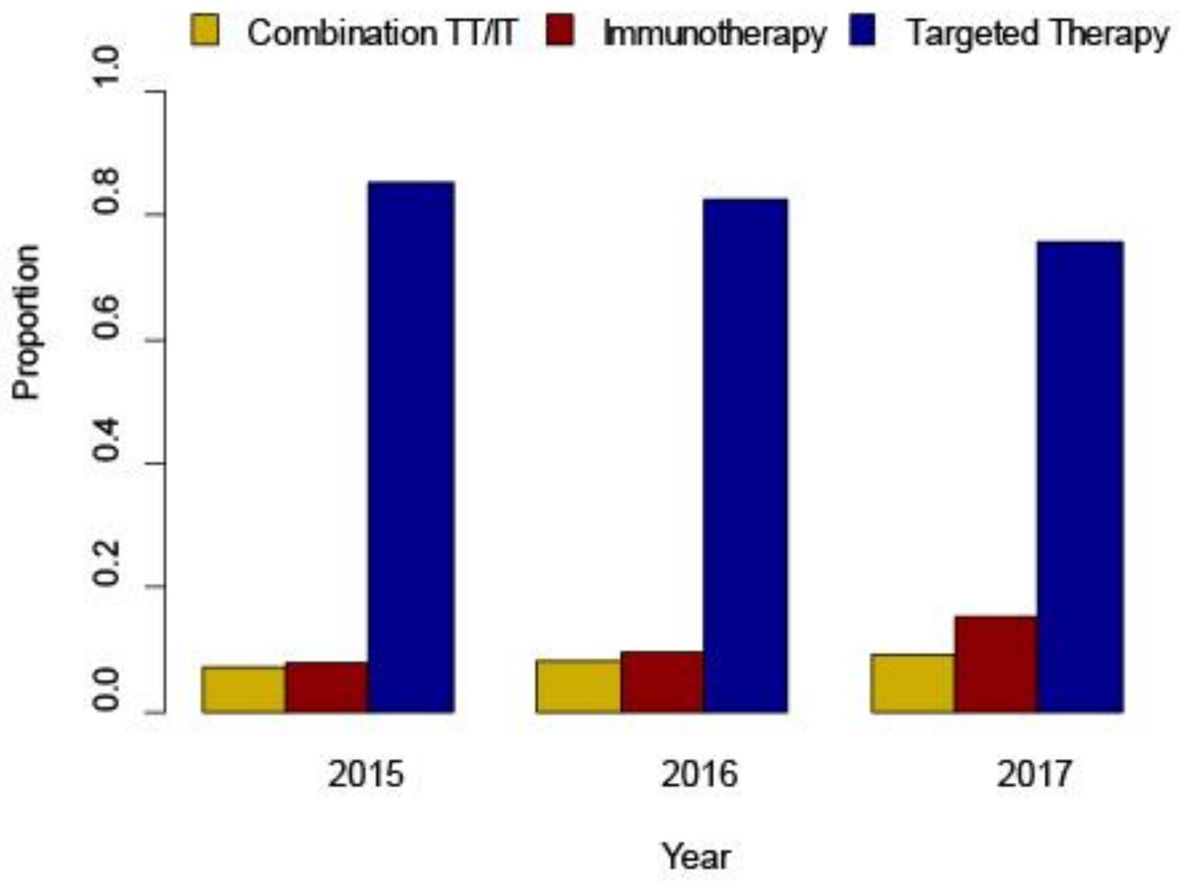

© 2021 Chakiryan NH et al. *JAMA Network Open.*
